# Sedentary behavior and motor competence in children and adolescents: a review

**DOI:** 10.11606/s1518-8787.2021055002917

**Published:** 2021-10-13

**Authors:** Guilherme dos Santos, Paulo Henrique Guerra, Suedem Andrade Milani, Ariane Brito Diniz Santos, Maria Teresa Cattuzzo, Alessandro Hervaldo Nicolai Ré

**Affiliations:** I Universidade de São Paulo Escola de Artes, Ciências e Humanidades Programa de Pós-Graduação em Ciências da Atividade Física São PauloSP Brasil Universidade de São Paulo. Escola de Artes, Ciências e Humanidades. Programa de Pós-Graduação em Ciências da Atividade Física. São Paulo, SP, Brasil; II Universidade Federal da Fronteira Sul ChapecóSC Brasil Universidade Federal da Fronteira Sul. Chapecó, SC, Brasil; III Universidade de Pernambuco Escola Superior de Educação Física RecifePE Brasil Universidade de Pernambuco. Escola Superior de Educação Física. Recife, PE, Brasil

**Keywords:** Child, Adolescent, Sedentary Behavior, Motor Competence, Systematic Review

## Abstract

**OBJECTIVE:**

To synthesize evidence from studies that analyzed the associations between sedentary behavior and motor competence in children and adolescents.

**METHODS:**

Systematic review of original articles that analyzed possible associations between sedentary behavior and motor competence in children and adolescents (3–18 years of age), without restrictions on study design, instruments and analysis protocols. The articles were identified through searches in the PubMed, *Web of Science, Academic Search Premier*, Cinahl, Medline and SPORTDiscus databases, as well as in reference lists. The level of evidence was evaluated according to the amount of studies that reported statistical significance in the associations between the variables and the quality of the articles (risk of bias).

**RESULTS:**

Of 2,462 initial studies, 22 composed the synthesis (two interventions, nine longitudinal and eleven cross-sectional studies). Of these, in 13, we observed negative associations between the variables, more often in the age group of seven to fourteen years. In the analysis of risk of bias, the main limitations of the studies were “convenience sampling” and “no description of sample sizing”.

**CONCLUSIONS:**

The available evidence suggests that sedentary behavior is negatively associated with motor competence in elementary school children, although the evidence is uncertain in the preschool years; the synthesis of results from longitudinal studies suggests that sedentary behavior negatively affects the development of motor competence. It is important that future studies have greater control over sociocultural determinants and deepen knowledge regarding sex and age, as well as the methods and indicators used to evaluate the two variables.

## INTRODUCTION

High sedentary behavior (SB) index, defined by activities with energy expenditure ≤ 1.5 metabolic equivalents (MET) while sitting or lying down during the waking period^1^, is currently a serious global public health problem^2,3^. SB during childhood and adolescence has been identified as a predictor of SB in adulthood^4,5^ and development of chronic diseases such as diabetes, hypertension and overweight or obesity^6^.

Several factors, such as access to technologies, the decrease in the supply of public space and rates of violence, have led children and adolescents to adopt a SB during much of their day^10^, particularly in leisure options involving screen activities (tablets, smartphones, computers, video games, television)^10,13,14^. Current guidelines suggest a daily limit of 1h in screen activities for the age group of 3 to 5 years and 2h daily from 5 to 17 years^15,16^. Despite these recommendations, studies^10,17,18^ have reported high screen time values (more than 3 to 4 hours per day) in children and adolescents of different nationalities, a fact probably aggravated due to the covid-19 pandemic^19^.

Along with the high rates of SB, a decline in motor competence (MC) has also been reported^20^, that is, competence in the execution of motor skills, with organization and movement control compatible with age^23^; this factor is potentially important to decrease SB indices and increase the practice of physical activity (PA)^12,24^. A growing body of evidence has indicated that MC favors participation in physical and sports activities^25^ and associates with better overall health outcomes, including adequate body weight and increased cardiorespiratory fitness^28^. Children and adolescents with low MC tend to avoid physical activity^12,17^ and adopt screen activities as leisure option^17,29^, which further restricts motor development and can generate a negative behavioral cycle, increasing the likelihood of physical inactivity and excessive SB throughout life. Recent research has found a negative association between SB and MC^12,30^ which suggests a reciprocal relationship between these variables^24^, with important application in health promotion policies.

However, in a meta-analysis article, the evidence of association between SB and MC was considered uncertain^31^ because, in addition to identifying only three surveys with children and adolescents^32^, they only found one^33^, conducted with children from 9 to 10 years of age, with significant association. Therefore, depending on the possible impact of SB guidelines on health policies^15^ and considering the importance of MC development in youth^23,24,28^, there is a need for a greater understanding of the association between MC and SB, considering a greater number of studies and the possible differences between age groups.

Thus, the objective of this systematic review was to synthesize the evidence of studies that analyzed the associations between SB and MC in children and adolescents.

## METHODS

### Protocol and Registration

This study is a systematic review, with its protocol registered in the International Prospective Register of Systematic Reviews (PROSPERO CRD42020161554). The full text was elaborated based on the items in the list Preferred Reporting Items of Systematic Reviews and Meta-Analyses (PRISM)^35^.

### Eligibility Criteria

Based on the research question, original articles published in peer-reviewed scientific journals in English, Portuguese and Spanish were sought. More specifically, other items were established from the PICO strategy^36^, considering:

### Population

Heterogeneous samples of children and adolescents (without specific deficiencies or clinical cases, except for samples specifically composed of overweight or obese children) aged 3 to 18 years. For our purposes, aiming to improve the degree of comparability and presentation of evidence, the subgroups were defined as follows, considering the Brazilian education system: preschoolers, from 3 to 6 years of age; elementary school, involving children and adolescents between 6 and 14 years; and high school, which covers adolescents between 15 and 18 years.

### Intervention or Exposure

Intervention studies were included that implemented strategies for the control or reduction of SB, regardless of the context (for example, at school or in the community) and characteristics (whether by theoretical, practical activities or both).

Observational studies analyzed possible associations between SB and MC, based on SB as an exposure variable and MC as an outcome variable. For the record, no restrictions were imposed on the types (e.g. screen time, sitting time), domains (e.g. leisure, school and travel) and instruments (e.g. questionnaires and motion sensors) used to measure SB or MC. Considering the current understanding of the concept, studies that addressed “sedentary” as absence of physical activity were excluded.

### Comparison

In the intervention studies, no restrictions were imposed on the existence or type of activities offered to the control groups, opting, when possible (as in studies with more than one control group), for comparators that received less activity load.

### Outcomes (Health Indicators)

The outcome was motor competence. To evaluate it, the gross motor skills of locomotion, object control and balance were considered.

### Study Design

Cross-sectional studies, cohorts and interventions were included that showed analyses on possible associations between SB and MC, regardless of the protocol used (e.g. univariate or multivariate analyses). Case studies, descriptive studies, reviews, meta-analyses, dissertations, theses and summaries of events were excluded.

### Sources of Information and Search Strategy

To recover potential studies, in March 2020, systematic searches were applied in six electronic databases: PubMed, Web of Science, Academic Search Premier, Cinahl, Medline and SPORTDiscus from the strategy applied in PubMed: ((((((((((motor competence[Text Word]) OR motor development[Text Word]) OR gross motor skills[Text Word]) OR fundamental motor skills[Text Word]) OR fundamental movement skills[Text Word]) OR motor coordination[Text Word]) OR motor ability[Text Word]) OR locomotor skills[Text Word]) OR object control skills[Text Word]) OR motor skills[Text Word]) AND (((((((sedentary behavior[Text Word]) OR sitting time[Text Word]) OR television[Text Word]) OR computer[Text Word]) OR videogame[Text Word]) OR screen time[Text Word]) OR screen activity*[Text Word]) AND child*[Text Word]. To avoid loss of relevant information, manual searches were conducted in the reference lists of articles evaluated by their full texts. No restrictions were imposed on the year of publication.

An author performed the initial search and entered all the recovered articles in the Rayyan platform (https://rayyan.qcri.org), where identification and removal of inter-database duplicates was conducted. Two authors (GS and SAM) independently reviewed the articles available by titles and abstracts. The results were compared and inconsistencies were discussed until a consensus was reached. If consensus was not reached, a third author (AHNR) would define the eligibility of the study. After this phase, the same authors evaluated the full texts of the remaining articles.

### Data Extraction

Data were extracted independently by two authors (GS and SAM), using an electronic spreadsheet, which was organized into two levels of information: (1) descriptive (location, design, sample and age) and (2) methodological (type and measurement of MC, type and measurement of SB, statistics and main results). In particular, the results related to the analysis between SB and MC were extracted, considering the positive, negative or null associations, according to magnitude and level of significance p < 0.05. Data were extracted independently by sex only if data from the total sample were unavailable. In the case of different results for each sex, the study was classified as uncertain association.

### Risk of Bias and Evaluation of the Quality of Studies

All included studies had their risk of bias assessed by two authors (GS and SAM), independently, with the support of the senior researcher (AHNR). For this purpose, the instrument developed by Lubans et al.^27^ was used, who, in turn, were based on the items of the STROBE and CONSORT guidelines. Scores of 0 (absent or inadequately described) or 1 (present and adequately described) were assigned in six questions, namely: (a) “Does the study describe the eligibility/selection criteria of participants?“; (b) “Were participants randomly selected?” ; (c) “Does the study mention sources and details of the MC assessment, and do these instruments have adequate reliability for this specific age group?”; (d) “Does the study mention sources and details of SB assessment, and do all methods have acceptable reliability?” ; (e) “Did the study report sample sizing and was it adequately sized to detect hypothetical relationships?”; (f) “Does the study mention the number of subjects who completed each of the different measurements, and did these participants complete at least 80% of the MC and SB measurements?”. It was previously established that studies with scores ≤ 2 would have high risk of bias; studies in the range between 3 and 4 points, medium risk of bias; and studies with scores between 5 and 6, low risk of bias.

### Summary of Results

Considering the heterogeneity between the designs and the methods adopted, since the first treatments, the construction of a descriptive synthesis of the available results was stipulated. The judgment of scientific evidence was based on Lubans et al.^27^ using the percentage of studies that reported a statistically significant association, while also considering the risk of bias: (a) lack of scientific evidence, if less than 33% of the studies indicate a significant association between the variables or none of the studies considered at low risk of bias find a significant association; (b) uncertain evidence, if 34 to 59% of the studies indicate a significant association between the variables; (c) positive (or negative) evidence, if 60 to 100% of the studies indicate a significant association between variables; (d) strong evidence, if 60 to 100% of the studies indicate a significant association between variables (in the same direction), there are no studies classified as uncertain association and more than 59% of the studies are considered to be at low risk of bias (score ≥ 5).

## RESULTS

**Figure 1:**
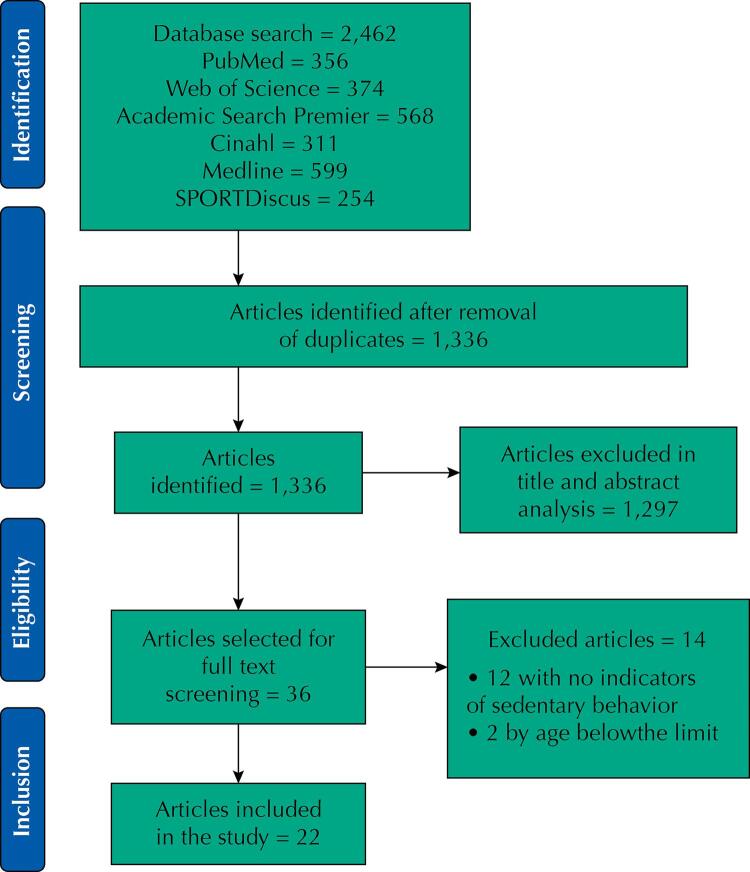
Flowchart of the systematic review.

The flow chart shows the selection process (Figure 1). In summary, of the 2,462 references initially identified, we evaluated 1,336 by titles and abstracts. Of these, we referred 36 for screening by full reading of the texts and excluded 14 because they did not show SB indicators (n = 12) or did not include the age group that was the target of our study (n = 2). Finally, we included 22 studies in the systematic review^12,17,30,32,33,37^.

Half of the studies included had a cross-sectional design (n = 11); also, there were nine longitudinal and two experimental studies (Table 1). The samples investigated included people aged 3 to 14 years. The sample size ranged from 17^49^ to 10,831^52^ participants. Most of the studies were conducted in North American countries (n = 9)^17,33,37,38,40,43^.

**Table 1:** Descriptive characteristic of the included studies.

Study	Local	Design	Sample	Age
Adank et al.^12^, 2018	Netherlands	Cross-sectional	595 (291 boys)	7–11 years
Barnett et al.^39^, 2012	Australia	Cross-sectional	53 (22 boys)	3–6 years
Burns et al.^38^, 2019	United States	Cross-sectional	409 (205 Boys)	1st to 5th year; mean of 8.4 years (SD = 1.8)
Cadoret et al.^40^, 2018	Canada	Longitudinal	133 (51 boys)	4–7 years
Capio et al.^41^, 2015	Hong Kong	Experimental	26 (13 boys)	Experimental group: mean of 7.17 years (SD = 2.77); control group: mean of 6.82 years (SD = 2.51)
Cliff et al.^32^, 2009	Australia	Cross-sectional	46 (25 boys)	3–5 years
Drenowatz and Cricket^30^, 2019	Austria	Longitudinal	213 (122 boys)	5th year; mean of 10.4 years (SD = 0.6)
Famelia et al.^42^, 2017	Indonesia	Cross-sectional	66 (30 boys)	3–6 years
Gu^43^, 2016	United States	Longitudinal	256 (129 boys)	5–6 years
Gu et al.^44^, 2018	United States	Longitudinal	141 (72 boys)	Preschoolers; mean 5.37 years (SD = 0.48)
Gu, Chen and Zhang^45^, 2019	United States	Longitudinal	671 (363 boys)	Mean of 6.96 years (SD = 1.60)
Jaakkola et al.^46^, 2009	Finland	Cross-sectional	152 (76 boys)	7th grade; 13 years
Jaakkola et al.^47^, 2019	Finland	Longitudinal	336 (173 boys)	6th year; mean of 12.02 years (SD = 0.38)
Laukkanen et al.^48^, 2014	Finland	Experimental	84 (38 boys)	5–8 years
Lloyd et al.^49^, 2014	Canada	Longitudinal	17 (5 boys)	6 years
Lopes et al.^33^, 2012	Portugal	Cross-sectional	213 (103 boys)	9–10 years
Lopes et al.^50^, 2016	Portugal	Cross-sectional	101 (53 boys)	3–5 years
Matarma et al.^51^, 2018	Finland	Longitudinal	111 (45 boys)	5–6 years
Smith et al.^52^, 2015	England	Longitudinal	10831 (N/A)	10 years
Tsuda et al.^53^, 2019	United States	Cross-sectional	72 (39 boys)	Preschoolers; mean of 4.38 years (SD = 0.85)
Webster et al.^17^, 2019	United States	Cross-sectional	126 (58 boys)	3-4 years
Wrotniak et al.^37^, 2006	United States	Cross-sectional	65 (31 boys)	8–10 years

SD: standard deviation.

Regarding the measures, fourteen studies used product measures, such as Bruininks-Oseretsky Test of Motor Proficiency Second Edition (BOT-2), Körperkoordinationstest für Kinder (KTK) and Movement Assessment Battery for Children Second Edition (MABC-2); to evaluate the MC, seven used process measures such as Test of Gross Motor Development (TGMD) - and only one study^17^ used both types. The TGMD (first, second or third version) was the most used motor test (n = 8)^17,38^. Three studies used the PE Metrics^43^, three studies used the BOT-2^37,40,51^, two studies used the KTK^33,48^ and two studies used the MABC-2^17,50^. Only one study used the Athletic Skills Track^12^ test and another used the Deutsche Motorik Test^30^. Three studies used specific motor tasks (e.g. walking backwards, balancing, throwing and grasping)^46,47,52^. Regarding SB measurement, sixteen studies used accelerometer as a direct measure and six studies used questionnaires. Regarding the questionnaires, five studies used them to evaluate recreational screen time and only one study used them to evaluate sitting time (Table 2).

**Table 2:** Methodological characteristic of the variables MC and SB, statistical analysis and main results.

Study	MC type and measurement	SB type and measurement	Statistics	Main results between SB and MC
Adank et al.^12^, 2018	Product; AST	Total time in SB; accelerometer	Multivariate analysis Very low MC and SB: β = 3.17; CI 1.28–5.05 Low MC and SB: β = 1.97; CI 0.44–3.49 High MC and SB: β = -0.45; CI-1.71–0.81 Very high MC and SB: β = -1.72; CI–3.18-0.27	Negative association
Barnett et al.^39^, 2012	Process; TGMD-2	Recreational screen time; questionnaire	Hierarchical linear regression, adjusted by age and sex Object and SB control skills: β = -0.13 Locomotion skills and SB: β = -0.24	Null association
Burns et al.^38^, 2019	Process; TGMD-3	Total time in SB; accelerometer	General linear models of mixed effects adjusted for age, body mass index, estimated aerobic capacity and school Locomotion skills and SB: γ = -9.07 Object control skills and SB: γ = 2.09 MC and SB: γ = 5.05	Null association
Cadoret et al.^40^, 2018	Product; BOT-2 SF	Recreational screen time; questionnaire	Pearson correlation SB at 4 years and MC at 7 years: r = -0.267 SB at 5 years and MC at 7 years: r = -0.268 SB at 7 years and MC at 7 years: r = -0.246	Negative association
Capio et al.^41^, 2015*	Process; TGMD-2	Total time in SB; accelerometer	Pearson correlation Locomotion skills and SB: r = -0.310 Object control skills and SB: r = -0.275 Running duration and SB: r = 0.603 Jumping distance and SB: r = -0.445 Kicking and SB: r = -0.411 Throwing and SB: r = 0.328 Grabbing and SB: r = -0.242	Negative association
Cliff et al.^32^, 2009	Process; TGMD-2	Total time in SB; accelerometer	Pearson correlation; MC and SB in boys: r = -0.194 MC and SB in girls: r = 0.138	Null association
Drenowatz and Cricket^30^, 2019	Product; DMT 6-18	Recreational screen time; questionnaire	Manova and Pearson correlation Increased media consumption with improvements in side jumps (p = 0.03) and decrease in 6-minute run (p = 0.03)	Negative association
Famelia et al.^42^, 2017	Process; TGMD-3	Total time in SB; accelerometer	Multiple regression Locomotion skills and SB during the playground: r = -0.56 Ball skills and SB during the playground: r = -0.14	Uncertain association
Gu^43^, 2016	Product; PE Metrics	Total time in SB; accelerometer	Pearson correlation Locomotion skills and SB: r = -0.13 Object control skills and SB: r = -0.16 MC and SB: r = -0.19	Negative association
Gu et al.^44^, 2018	Product; PE Metrics	Total time in SB; accelerometer	Pearson correlation Locomotion skills and SB: r = -0.30 Object control skills and SB: r = -0.30 MC and SB: r = -0.34	Negative association
Gu, Chen and Zhang^45^, 2019	Product; PE Metrics	Total time in SB; accelerometer	Pearson correlation Locomotion skills and SB in Hispanics: r = -0.25 Object control skills and SB in Hispanics: r = -0.08 Locomotion skills and SB in non-Hispanics: r = -0.16 Object control skills and SB in non-Hispanics: r = -0.06	Negative association
Jaakkola et al.^46^, 2009	Product; throwing, jumping and balance	Recreational screen time; questionnaire	Pearson correlation Throwing and SB: r = 0.09 Jumping and SB: r = -0.28 Balance and SB: r = 0.22	Negative association
Jaakkola et al.^47^, 2019	Product; Five jumps, throwing and grasping	Total time in SB; accelerometer	Structural equation modeling Grade 6: Locomotion skills and SB: r = -0.056 Object control skills and SB: r = -0.142 7th grade: Locomotion skills and SB: r = -0.364 Object control skills and SB: r = -0.059	Uncertain association
Laukkanen et al.^48^, 2014	Product; KTK and TCB	Total time in SB; accelerometer	Partial correlation MC and SB in preschool boys: r = -0.52	Uncertain association
Lloyd et al.^49^, 2014	Process; TGMD	Sitting time; questionnaire	Pearson correlation Locomotion skills and SB: r = -0.37 Object control skills and SB: -0.10 MC and SB: r = -0,25	Negative association
Lopes et al.^33^, 2012	Product; KTK	Total time in SB; accelerometer	Binary logistic regression OR = 5.065 for girls and OR = 9.149 for boys	Negative association
Lopes et al.^50^, 2016	Product; MABC-2	Total time in SB; accelerometer	Spearman correlation Balance and SB: r = 0.15 Object control skills and SB: r = 0.03	Null association
Matarma et al.^51^, 2018	Product; BOT-2	Total time in SB; accelerometer	Linear regression No significant correlation	Null association
Smith et al.^52^, 2015	Product; Throwing, balance on one foot, walking backwards	Recreational screen time; questionnaire	Logistic regression High MC and low probability of high screen time at 16 years: OR = 0.79; CI 0.64–0.98 High MC and low probability of TV time at age 42: OD = 0.85; CI 0.72–0.99	Negative association
Tsuda et al.^53^, 2019	Process; TGMD-2	Total time in SB; accelerometer	Pearson correlation Locomotion skills and SB: r = -0.46 Object control skills and SB: r = -0.42	Negative association
Webster et al.^17^, 2019	Process and product; TGMD - 3 and MABC-2	Total time in SB; accelerometer	Pearson correlation MC and screen time: β = -1.6	Null association
Wrotniak et al.^37^, 2006	Product; BOTMP-SF	Total time in SB; accelerometer	Pearson correlation MC and SB: r = -0,308	Negative association

MC: motor competence; SB: sedentary behavior; OD: odds ratio; CI: confidence interval; Manova: multivariate analysis of variance; AST: Athletic Skills Track; BOT-2: Bruininks-Oseretsky Test of Motor Proficiency Second Edition; BOT-2 SF: Bruininks-Oseretsky Test of Motor Proficiency Second Edition – Short Form; BOTMP-SF: Bruininks-Oseretsky Test of Motor Proficiency – Short Form; DMT 6–18: *Deutsche Motorik Test*; KTK: *Körperkoordinationstest für Kinder*; MABC-2: Movement Assessment Battery for Children Second Edition; TCB: Underarmor Throw and Catch a Ball; TGMD: Test of Gross Motor Development [second and third editions marked by the number after the acronym].

Note: only data from children with typical development were considered for the results.

**Figure 2:**
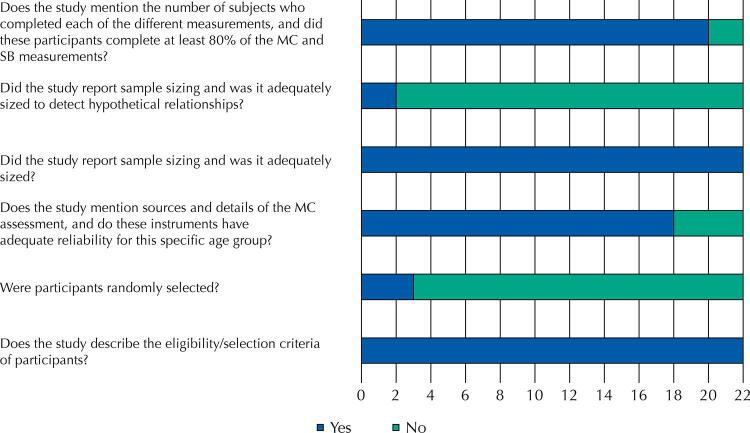
Analysis by risk of bias of the included articles.

As for the risk of bias, 18.2% (n = 4) of the studies obtained a low-risk score (≥ 5), 77.3% (n = 17) obtained a medium risk score, and only one study obtained a high risk score. All studies met the criteria: (a) “Does the study describe the eligibility/selection criteria of participants?“ and (d) “Does the study mention sources and details of the SB assessment?”. Finally, the most absent quality items were: (b) “Were participants randomly selected?” and (e) Did the study report sample sizing and was it properly sized to detect hypothetical relationships?” (Figure 2).

Of the total studies investigated (n = 22), thirteen indicated negative associations between SB and MC, six did not indicate association and three indicated uncertain associations.

In the stratum of studies evaluated with low risk of bias (n = 4), a negative association was found in three studies, highlighting as main results: SB and high MC in schoolchildren^12^; SB and MC in preschoolers^44^; MC in childhood and sitting time after 20 years^49^. Studies with moderate risk of bias also showed negative association results^30,33,37,40,41,43,45,46,52,53^.

Considering experimental studies (n = 2) of medium risk of bias, a study^41^ found a negative association between SB and MC in the ability to move and control objects, but the other^48^ found uncertain association. Considering the longitudinal studies (n = 9), two studies with low risk of bias found a negative association between SB and MC^44,49^, and five other studies with medium risk of bias^30,40,43,45,52^ also found a negative association between SB and MC. In contrast, a study of high risk of bias^51^ found no association and a study of moderate risk of bias^47^ found uncertain association. Considering the cross-sectional studies (n = 11) a study of low risk of bias found a negative association between SB and MC^12^ and four studies of medium risk of bias^33,37,46,53^ found a negative association between SB and MC. In contrast, a study of low risk of bias^39^ and four studies of medium risk of bias^17,32,38,50^ found no association, and a study of medium risk of bias^42^ found uncertain association.

Analyzing the age group of preschoolers (n = 11), two studies of low risk of bias^44,49^ and three studies of medium risk of bias^40,43,53^ found a negative association between SB and MC. In contrast, a study of low risk of bias^39^, three studies of medium risk of bias^17,32,50^ and one study of high risk of bias^51^ found no association, while one study found uncertain association^42^. Therefore, according to the established criteria, the results show uncertain evidence of association between SB and MC in this age group. Considering the age group of elementary school (n = 11), a study of low risk of bias^12^ and seven studies^30,33,37,41,45,46,52^ of medium risk of bias found negative association between SB and MC. In contrast, a study of medium risk of bias^38^ found no association and two studies found uncertain association. Therefore, the results indicate evidence of a negative association between SB and MC in this age group (Table 3).

**Table 3:** Distribution of studies that investigated sedentary behavior and motor competence by the risk of bias within the studies and by the level of scientific evidence.

Education	Studies that show association	Studies by risk of bias	Association or non-association according to the risk of bias	Level of evidence
Preschool (n = 11)	Negative association: 5 (45.4%) No association: 5 (45.4%) Uncertain association: 1 (9.1%)	Low: 3 (27.2%) Medium: 7 (63.6%) High: 1 (9.0%)	LRB: N:^44,49^; NA:^39^ MRB: N:^40,43,53^; NA:^32,50,17^; ?: ^42^ HRB: NA:^51^	Uncertain evidence
Primary education (n = 11)	Negative association: 8 (88.8%) No association: 1 (9.0%) Uncertain association: 2 (18.1)	Low: 1 (9.0%) Average: 10 (90.9%)	LRB: N:^12^ MRB: N:^41,30,45,46,33,52,37^; NA:^38^; ?:^47,49^	Evidence of negative association

LRB: low risk of bias; MRB: medium risk of bias; HRB: high risk of bias; N: negative association; NA: null association; ?: uncertain association.

In short, considering the result of the associations and the risk of bias, there was scientific evidence for the negative association between SB and MC of children and adolescents in elementary school and uncertain scientific evidence for association in preschool children.

## DISCUSSION

This review synthesized the results of studies that evaluated associations between SB and MC in childhood and adolescence. According to the results, there is evidence for negative association between SB and MC in elementary school years. Even though most of the studies examined used a cross-sectional design, making inferences about causality difficult, seven longitudinal studies pointed to negative associations between SB and MC, suggesting that time in SB may impair the development of MC^30,40,43^.

A meta-analysis by Engel et al.^54^ suggests that MC and PA levels may increase through interventions in childhood, corroborating the findings of a previous review^55^, in which object control skills were strongly associated with PA in boys, and locomotion skills were associated with PA in girls. A recent longitudinal study demonstrated decreased PA and increased SB between 6 and 11 years^56^. A plausible hypothesis, based on the results found in this study and the conceptual model proposed by Stodden et al.^24^, is that the adequate MC could influence the increase of PA and the decrease of SB. It is possible that children with excess SB have few opportunities for developing MC^30^, further increasing the likelihood of adoption of SB (screen activities) in leisure moments^18,29^ and generating a vicious behavioral cycle with unhealthy lifestyle habits associated with the emergence of chronic-degenerative diseases^24^. Thus, it is important that public health policies consider the reciprocal and dynamic relationship between SB and MC and promote, simultaneously, the improvement of MC and the decrease of SB, particularly leisure time in screen activities during childhood and adolescence.

As for environmental factors, the theoretical model by Hulteen et al.^57^ suggests that motor skills vary in importance and popularity according to the cultural and geographical context, with an important role for the maintenance of PA throughout life. Considering the role of physical activity as a form of intervention to decrease SB and contribute to the development of MC, it is possible to suggest that the higher the MC in different abilities (for example, kicking, throwing, bouncing and jumping), the greater the range of possibilities of PA practice that could replace SB. The development of MC in skills that predominate in the cultural and geographical context in question can be an effective strategy to replace SB with a PA that the child or adolescent has the competence to perform. Reinforcing this perspective, data from a Finnish study^41^ point out a negative association between kicking skills and SB, being soccer one of the predominant youth sports in the country^58^.

Data from this review reinforce the importance of integrated public education and health policies focused simultaneously on improving MC and decreasing SB, particularly screen time at leisure. Interventions in children who spend excessive time in SB should include the development of MC continuously, that is, they should consider the quality of the practice of PA, not only the amount of time spent in this practice^59,60^. Improvement of MC may be a promising strategy to reduce SB and increase moderate to vigorous PA, especially for children with low MC^12^. Longitudinal evaluations should strengthen future research to provide a better understanding of causality between SB and MC.

In addition, there is a wide scope to be explored, considering environmental factors, gender and assessment methods, as well as the gap in the transition between age groups (children, adolescents, young adults and older adults). It is plausible that the causal direction between the variables is influenced by the age group. We suggest that future studies use two or more motor tests^22,61^ and the use of the accelerometer in conjunction with the questionnaire for a better understanding of SB and environmental factors. We also suggest randomized selection of participants and description of the statistical power of the study. Finally, it is also important to highlight that the results found are limited to adolescents up to 14 years, providing a wide field of research among adolescents and young adults in this topic.

This study contains some limitations. Although the search was comprehensive, we could only include studies in the English language. The bias related to the selective reporting of associations in the studies may be a possibility, and adjustments in the associations between SB and MC were not considered as a function of the practice of PA. In addition, limitations in the evidence base influenced the results of this review. The practice of moderate to vigorous PA (MVPA) may partially offset the negative effects of SB^31,62^, being independent behaviors, i.e. high levels of SB do not necessarily imply low levels of MVPA and vice versa; people who meet the recommendations of MVPA (≥ 1h daily) may still have SB at many hours of the day^63^. In reality, to date, SB recommendations are restricted to screen time^1,15,64^. From a mathematical point of view, if the recommendation of at least three hours a day of PA at any intensity (including 1h of MVPA) is applied^1,15^, and considering a period of 12 hours of wakefulness, the time limit of SB would be 9 hours daily. Therefore, a greater understanding of the context in which SB manifests itself and its association with the overall development of the child or adolescent is necessary.

Another limitation is the heterogeneity in the forms used to evaluate SB^65^ (questionnaires with information about total screen time or only TV/computer time or sitting time and direct accelerometry measurements), making it impossible to differentiate SB in screen/leisure activities and SB in other domains, such as sitting time in displacement or studies. The use of different motor tests to measure MC is also an important limitation. In total, at least 10 different MC evaluations were used. In literature, measures oriented to the process or product of movement can evaluate MC, and this can influence the magnitude of the associations^22^.

Finally, the results of this systematic review demonstrate that there is evidence of a negative association between SB and MC in children and adolescents in the elementary school period, although the evidence is uncertain in the preschool years. Environmental factors, as well as the method of assessment and age group, can be determinants for a better understanding of the investigated phenomenon. To better understand the associations between SB and MC, we suggest the establishment of standardized criteria for conducting studies, highlighting the context in which SB manifests itself (for example, in leisure situations or school activities). Improving the predominant MC in the sociocultural context in which the person is inserted can contribute to the decrease of SB and promote engagement in an active lifestyle in the long term for children and adolescents.
