# Supplementary material for: Sedentary behavior and motor competence in children and adolescents: a review
Source: Rev Saude Publica. 2021 Oct 13;55:57. doi: 10.11606/s1518-8787.2021055002917 (PMC8522757; doi:10.11606/s1518-8787.2021055002917)
Supplement: Translation

# Translation: Comportamento sedentário e competência motora em crianças e adolescentes: revisão

**Authors:** Guilherme dos Santos, Paulo Henrique Guerra, Suedem Andrade Milani, Ariane Brito Diniz Santos, Maria Teresa Cattuzzo, Alessandro Hervaldo Nicolai Ré

**Affiliations:** I Brasil Universidade de São Paulo. Escola de Artes, Ciências e Humanidades. Programa de Pós-Graduação em Ciências da Atividade Física. São Paulo, SP, Brasil; II Brasil Universidade Federal da Fronteira Sul. Chapecó, SC, Brasil; III Brasil Universidade de Pernambuco. Escola Superior de Educação Física. Recife, PE, Brasil

**Keywords:** Criança, Adolescente, Comportamento Sedentário, Competência Motora, Revisão Sistemática

## Abstract

**OBJETIVO:**

Sintetizar as evidências de estudos que analisaram as associações entre comportamento sedentário e competência motora em crianças e adolescentes.

**MÉTODOS:**

Revisão sistemática de artigos originais que analisaram possíveis associações entre comportamento sedentário e competência motora em crianças e adolescentes (3–18 anos de idade), sem restrições quanto ao delineamento de estudo, instrumentos e protocolos de análise. Os artigos foram identificados por meio de buscas nas bases PubMed, *Web of Science, Academic Search Premier*, Cinahl, Medline e SPORTDiscus, assim como em listas de referências. O nível de evidência foi avaliado de acordo com a quantidade de estudos que reportaram significância estatística nas associações entre as variáveis e a qualidade dos artigos (o risco de viés).

**RESULTADOS:**

De 2.462 estudos iniciais, 22 compuseram a síntese (duas intervenções, nove longitudinais e onze transversais). Desses, em 13 foram observadas associações negativas entre as variáveis, mais frequentemente na faixa etária de sete a catorze anos. Na análise do risco de viés, as principais limitações dos estudos foram “amostragens por conveniência” e “não descrição do dimensionamento amostral”.

**CONCLUSÕES:**

A evidência disponível sugere que o comportamento sedentário está negativamente associado à competência motora em crianças do ensino fundamental, embora a evidência seja incerta nos anos pré-escolares; a síntese de resultados dos estudos longitudinais sugere que o comportamento sedentário afeta negativamente o desenvolvimento da competência motora. É importante que futuros estudos tenham maior controle sobre os determinantes socioculturais e aprofundem os conhecimentos em relação ao sexo e à faixa etária, assim como dos métodos e indicadores utilizados para avaliação das duas variáveis.

## INTRODUÇÃO

O elevado índice de comportamento sedentário (CS), definido por atividades com gasto energético ≤ 1,5 equivalentes metabólicos (MET) enquanto sentado ou deitado durante o período de vigília^1^, é atualmente um grave problema de saúde pública mundial^2,3^. O CS durante a infância e adolescência tem sido identificado como um preditor do CS na idade adulta^4,5^ e desenvolvimento de doenças crônicas como diabetes, hipertensão e sobrepeso ou obesidade^6^.

Diversos fatores, como o acesso às tecnologias, a diminuição da oferta de espaço público e os índices de violência, têm levado crianças e adolescentes a adotar um CS durante grande parte de seu dia^10^, particularmente em opções de lazer que envolvem atividades de tela (tablets, smartphones, computadores, videogames, televisão)^10,13,14^. Diretrizes atuais sugerem um limite diário de 1h em atividades de tela para a faixa etária de 3 a 5 anos e de 2h diárias dos 5 aos 17 anos^15,16^. Apesar dessas recomendações, têm sido reportado valores elevados de tempo de tela (mais de 3 a 4h por dia) em crianças e adolescentes de diferentes nacionalidades^10,17,18^, fato provavelmente agravado em função da pandemia de covid-19^19^.

Juntamente com os altos índices de CS, também tem sido reportado um declínio na competência motora (CM)^20^, ou seja, competência na execução de habilidades motoras, com organização e controle de movimentos compatível com a idade^23^; esse fator é potencialmente importante para a diminuição nos índices de CS e aumento da prática de atividade física (AF)^12,24^. Um crescente corpo de evidências tem indicado que a CM favorece a participação em atividades físicas e esportivas^25^ e está associada a melhores resultados gerais de saúde, incluindo um peso corporal adequado e maior aptidão cardiorrespiratória^28^. Crianças e adolescentes com baixa CM tendem a evitar a prática de atividade física^12,17^ e adotar atividades de tela como opção de lazer^17,29^, o que restringe ainda mais o desenvolvimento motor e pode gerar um ciclo comportamental negativo, aumentando a probabilidade de inatividade física e CS excessivo ao longo da vida. Pesquisas recentes têm encontrado uma associação negativa entre CS e CM^12,30^ que sugere uma relação recíproca entre essas variáveis^24^, com importante aplicação em políticas de promoção da saúde.

Entretanto, em artigo de metanálise, a evidência de associação entre CS e CM foi considerada incerta^31^ pois, além de terem sido identificadas apenas três pesquisas com crianças e adolescentes^32^, em somente uma delas^33^, realizada com crianças de 9 a 10 anos de idade, houve associação significante. Portanto, em função do possível impacto das diretrizes de CS em políticas de saúde^15^ e considerando a importância do desenvolvimento da CM na juventude^23,24,28^, existe a necessidade de um maior entendimento da associação entre CM e CS, levando em conta um maior número de pesquisas e as possíveis diferenças entre faixas etárias.

Assim, o objetivo desta revisão sistemática foi sintetizar as evidências de estudos que analisaram as associações entre CS e CM em crianças e adolescentes.

## MÉTODOS

### Protocolo e Registro

O presente estudo configura-se como uma revisão sistemática, tendo seu protocolo registrado no *International Prospective Register of Systematic Reviews* (PROSPERO CRD42020161554). O texto integral foi elaborado com base nos itens da lista *Preferred Reporting Items of Systematic Reviews and Meta-Analyses* (PRISMA)^35^.

### Critérios de Elegibilidade

Com base na questão de pesquisa, foram procurados artigos originais publicados em revistas científicas com avaliação por pares nos idiomas inglês, português e espanhol. Mais especificamente, outros itens foram estabelecidos a partir da estratégia “Picos”^36^, considerando:

### População

Amostras heterogêneas de crianças e adolescentes (sem deficiências nem quadros clínicos específicos, à exceção de amostras especificamente compostas por crianças com sobrepeso ou obesidade) com idade entre 3 e 18 anos. Para nossos fins, visando melhorar o grau de comparabilidade e apresentação da evidência, os subgrupos foram definidos da seguinte forma, considerando o sistema de educação brasileiro: pré-escolares, dos 3 aos 6 anos de idade; ensino fundamental, envolvendo crianças e adolescentes entre 6 e 14 anos; e ensino médio, que abrange adolescentes entre 15 e 18 anos.

### Intervenção ou Exposição

Foram incluídos estudos de intervenção que implementaram estratégias para o controle ou redução do CS, independentemente do contexto (por exemplo, na escola ou na comunidade) e características (seja por atividades teóricas, práticas ou ambas).

Já os estudos observacionais analisaram possíveis associações entre o CS e a CM, tomando como base o CS enquanto variável de exposição e a CM enquanto variável de desfecho. Para constar, não foram impostas restrições relacionadas aos tipos (ex.: tempo de tela, tempo sentado), domínios (ex.: lazer, escola e deslocamento) e instrumentos (ex.: questionários e sensores de movimento) utilizados para a mensuração do CS, assim como para a CM. Considerando o atual entendimento do conceito, foram excluídos os estudos que abordaram “sedentário” enquanto ausência de atividade física.

### Comparação

Nos estudos de intervenção, não foram impostas restrições quanto à existência ou tipo de atividades oferecidas aos grupos controle, optando-se, quando possível (como em estudos com mais de um grupo controle), por comparadores que receberam menor carga de atividade.

### Desfechos (Indicadores de Saúde)

O desfecho foi a competência motora. Para avaliá-la, foram consideradas as habilidades motoras grossas de locomoção, controle de objetos e equilíbrio.

### Delineamento do Estudo

Foram incluídos estudos transversais, coortes e intervenções que apresentassem análises sobre possíveis associações entre o CS e a CM, independentemente do protocolo utilizado (ex.: análises univariadas ou multivariadas). Estudos de caso, descritivos, revisões, metanálises, dissertações, teses e resumos de eventos foram excluídos.

### Fontes de Informação e Estratégia de Busca

Para recuperação dos estudos potenciais, em março de 2020, buscas sistemáticas foram aplicadas em seis bases de dados eletrônicas: PubMed , *Web of Science, Academic Search Premier*, Cinahl, Medline e SPORTDiscus, partindo da estratégia aplicada no PubMed: ((((((((((motor competence[Text Word]) OR motor development[Text Word]) OR gross motor skills[Text Word]) OR fundamental motor skills[Text Word]) OR fundamental movement skills[Text Word]) OR motor coordination[Text Word]) OR motor ability[Text Word]) OR locomotor skills[Text Word]) OR object control skills[Text Word]) OR motor skills[Text Word]) AND (((((((sedentary behavior[Text Word]) OR sitting time[Text Word]) OR television[Text Word]) OR computer[Text Word]) OR videogame[Text Word]) OR screen time[Text Word]) OR screen activit*[Text Word]) AND child*[Text Word]. Para evitar perda de informação relevante, buscas manuais foram conduzidas nas listas de referências dos artigos avaliados pelos seus textos integrais. Não foram impostas restrições em relação ao ano de publicação.

Um autor realizou a busca inicial e introduziu todos os artigos recuperados na plataforma Rayyan (https://rayyan.qcri.org), onde foi conduzida identificação e remoção das duplicatas interbases de dados. Dois autores (GS e SAM), de forma independente, revisaram os artigos disponíveis pelos títulos e resumos. Os resultados foram comparados e as inconsistências foram discutidas até se alcançar um consenso. Caso não se chegasse ao consenso, um terceiro autor (AHNR) definiria a elegibilidade do estudo. Terminada essa fase, os mesmos autores avaliaram os textos completos dos artigos remanescentes.

### Extração de Dados

Os dados foram extraídos de forma independente por dois autores (GS e SAM), utilizando uma planilha eletrônica, que foi organizada em dois níveis de informações: (1) descritivas (local, desenho, amostra e idade) e (2) metodológicas (tipo e medida de CM, tipo e medida de CS, estatística e principais resultados). Em particular, foram extraídos os resultados relacionados às análises entre CS e CM, considerando as associações positivas, negativas ou nulas, de acordo com magnitude e nível de significância p < 0,05. Os dados foram extraídos independentemente por sexo apenas se os dados da amostra total não estivessem disponíveis. No caso de resultados diferentes para cada sexo, o estudo foi classificado como de associação incerta.

### Risco de Viés e Avaliação da Qualidade dos Estudos

Todos os estudos incluídos tiveram seu risco de viés avaliado por dois autores (GS e SAM), de forma independente, com apoio do pesquisador sênior (AHNR). Para tanto, foi utilizado o instrumento desenvolvido por Lubans et al.^27^, que, por sua vez, basearam-se nos itens das diretrizes STROBE e CONSORT. Foram atribuídas pontuações de 0 (ausente ou inadequadamente descrito) ou 1 (presente e descrito adequadamente) em seis questões, a saber: (a) “O estudo descreve os critérios de elegibilidade/seleção dos participantes?”; (b) “Os participantes foram selecionados aleatoriamente?”; (c) “O estudo menciona fontes e detalhes da avaliação da CM, e esses instrumentos têm confiabilidade adequada para essa faixa etária específica?”; (d) “O estudo menciona fontes e detalhes da avaliação de CS, e todos os métodos têm confiabilidade aceitável?”; (e) O estudo relatou dimensionamento amostral e foi adequadamente dimensionado para detectar relações hipotéticas?”; (f) “O estudo menciona o número de sujeitos que completaram cada uma das diferentes medidas, e esses participantes concluíram pelo menos 80% das medidas de CM e CS?”. Estabeleceu-se previamente que estudos com pontuação ≤ 2 teriam alto risco de viés; estudos na faixa entre 3 e 4 pontos, médio risco de viés; e estudos com pontuação entre 5 e 6, baixo risco de viés.

### Síntese de Resultados

Considerando a heterogeneidade entre os delineamentos e os métodos adotados, desde as primeiras tratativas, estipulou-se a construção de uma síntese descritiva dos resultados disponíveis. O julgamento da evidência científica foi baseado em Lubans et al.^27^, usando a porcentagem de estudos que reportaram significância estatística na associação, mas também levando em consideração o risco de viés: (a) falta de evidência científica, se menos de 33% dos estudos indicarem associação significativa entre as variáveis ou nenhum dos estudos considerados de baixo risco de viés encontrarem uma associação significativa; (b) evidência incerta, se 34 a 59% dos estudos indicarem uma associação significativa entre as variáveis; (c) evidência positiva (ou negativa), se 60 a 100% dos estudos indicarem uma significativa associação entre variáveis; (d) forte evidência, se 60 a 100% dos estudos indicarem uma associação significativa entre variáveis (na mesma direção), não houver estudos classificados como associação incerta e mais de 59% dos estudos forem considerados de baixo risco de viés (pontuação ≥ 5).

## RESULTADOS

O fluxograma apresenta o processo de seleção (Figura 1). De forma resumida, das 2.462 referências inicialmente identificadas, 1.336 foram avaliadas pelos títulos e resumos. Dessas, 36 foram encaminhadas para triagem por leitura integral dos textos e 14 foram excluídas por não apresentar indicadores de CS (n = 12) ou não incluir a faixa etária que era alvo do presente estudo (n = 2). Finalmente, 22 estudos foram incluídos na revisão sistemática^12,17,30,32,33,37^.

**Figura 1:** Fluxograma da revisão sistemática.

A metade dos estudos incluídos apresentou desenho transversal (n = 11), destacando-se também a presença de nove estudos longitudinais e dois experimentais (Tabela 1). As amostras investigadas incluíam pessoas de 3 a 14 anos de idade. O tamanho da amostra variou de 17^49^ a 10.831^52^ participantes. A maior parte dos estudos foi conduzida nos países da América do Norte (n = 9)^17,33,37,38,40,43^.

**Tabela 1:** Característica descritiva dos estudos incluídos.

Estudo	Local	Delineamento	Amostra	Idade
Adank et al.^12^, 2018	Holanda	Transversal	595 (291 meninos)	7–11 anos
Barnett et al.^39^, 2012	Austrália	Transversal	53 (22 meninos)	3–6 anos
Burns et al.^38^, 2019	Estados Unidos	Transversal	409 (205 meninos)	1º ao 5º ano; média de 8,4 anos (DP = 1,8)
Cadoret et al.^40^, 2018	Canadá	Longitudinal	133 (51 meninos)	4–7 anos
Capio et al.^41^, 2015	Hong Kong	Experimental	26 (13 meninos)	Grupo experimental: média de 7,17 anos (DP = 2,77); grupo controle: média de 6,82 anos (DP = 2,51)
Cliff et al.^32^, 2009	Austrália	Transversal	46 (25 meninos)	3–5 anos
Drenowatz e Greier^30^, 2019	Áustria	Longitudinal	213 (122 meninos)	5º ano; média de 10,4 anos (DP = 0,6)
Famelia et al.^42^, 2017	Indonésia	Transversal	66 (30 meninos)	3–6 anos
Gu^43^, 2016	Estados Unidos	Longitudinal	256 (129 meninos)	5–6 anos
Gu et al.^44^, 2018	Estados Unidos	Longitudinal	141 (72 meninos)	Pré-escolares; média de 5,37 anos (DP = 0,48)
Gu, Chen e Zhang^45^, 2019	Estados Unidos	Longitudinal	671 (363 meninos)	Média de 6,96 anos (DP = 1,60)
Jaakkola et al.^46^, 2009	Finlândia	Transversal	152 (76 meninos)	7º ano; 13 anos
Jaakkola et al.^47^, 2019	Finlândia	Longitudinal	336 (173 meninos)	6º ano; média de 12,02 anos (DP = 0,38)
Laukkanen et al.^48^, 2014	Finlândia	Experimental	84 (38 meninos)	5–8 anos
Lloyd et al.^49^, 2014	Canadá	Longitudinal	17 (5 meninos)	6 anos
Lopes et al.^33^, 2012	Portugal	Transversal	213 (103 meninos)	9–10 anos
Lopes et al.^50^, 2016	Portugal	Transversal	101 (53 meninos)	3–5 anos
Matarma et al.^51^, 2018	Finlândia	Longitudinal	111 (45 meninos)	5–6 anos
Smith et al.^52^, 2015	Inglaterra	Longitudinal	10831 (N/A)	10 anos
Tsuda et al.^53^, 2019	Estados Unidos	Transversal	72 (39 meninos)	Pré–escolares; média de 4,38 anos (DP = 0,85)
Webster et al.^17^, 2019	Estados Unidos	Transversal	126 (58 meninos)	3–4 anos
Wrotniak et al.^37^, 2006	Estados Unidos	Transversal	65 (31 meninos)	8–10 anos

DP: desvio-padrão.

Em relação às medidas, catorze estudos usaram medidas de produto – como *Bruininks–Oseretsky Test of Motor Proficiency Second Edition* (BOT-2), *Körperkoordinationstest für Kinder* (KTK) e *Movement Assessment Battery for Children Second Edition* (MABC-2) – para avaliar a CM, sete utilizaram medidas de processo – como o *Test of Gross Motor Development* (TGMD) – e apenas um estudo^17^ utilizou ambos os tipos. O TGMD (primeira, segunda ou terceira versão) foi o teste motor mais utilizado (n = 8)^17,38^. Três estudos utilizaram o *PE Metrics*^43^, três estudos utilizaram o BOT-2 ^37,40,51^, dois estudos utilizaram o KTK^33,48^ e dois estudos utilizaram o MABC-2^17,50^. Os testes *Athletic Skills Track*^12^ e *Deutsche Motorik Test*^30^, foram utilizados em um estudo. Três estudos utilizaram tarefas motoras específicas (ex.: andar para trás, equilíbrio, arremessar e agarrar)^46,47,52^. Com relação à medida de CS, dezesseis estudos utilizaram acelerômetro como medida direta e seis estudos fizeram uso de questionários. Com relação aos questionários, cinco estudos os utilizaram para avaliar tempo de tela recreativo e apenas um estudo os utilizou para avaliar tempo sentado (Tabela 2).

**Tabela 2:** Característica metodológica das variáveis CM e CS, análise estatística e principais resultados.

Estudo	Tipo e medida de CM	Tipo e medida de CS	Estatística	Principais resultados entre CS e CM
Adank et al.^12^, 2018	Produto; AST	Tempo total em CS; acelerômetro	Análise multivariada Muito baixa CM e CS: β = 3,17; IC 1,28–5,05 Baixa CM e CS: β = 1,97; IC 0,44–3,49 Alta CM e CS: β = −0,45; IC −1,71–0,81 Muito alta CM e CS: β = −1,72; IC −3,18–0,27	Associação negativa
Barnett et al.^39^, 2012	Processo; TGMD-2	Tempo de tela recreativo; questionário	Regressão linear hierárquica, ajustada por idade e sexo Habilidades de controle de objeto e CS: β = −0,13 Habilidades de locomoção e CS: β = −0,24	Associação nula
Burns et al.^38^, 2019	Processo; TGMD-3	Tempo total em CS; acelerômetro	Modelos lineares gerais de efeitos mistos ajustados à idade, índice de massa corporal, capacidade aeróbica estimada e escola Habilidades de locomoção e CS: γ = −9,07 Habilidades de controle de objetos e CS: γ = 2,09 CM e CS: γ = 5,05	Associação nula
Cadoret et al.^40^, 2018	Produto; BOT-2 SF	Tempo de tela recreativo; questionário	Correlação de Pearson CS aos 4 anos e CM aos 7 anos: r = −0,267 CS aos 5 anos e CM aos 7 anos: r = −0,268 CS aos 7 anos e CM aos 7 anos: r = −0,246	Associação negativa
Capio et al.^41^, 2015*	Processo; TGMD-2	Tempo total em CS; acelerômetro	Correlação de Pearson Habilidades de locomoção e CS: r = −0,310 Habilidades de controle de objetos e CS: r = −0,275 Duração da corrida e CS: r = 0,603 Distância do salto e CS: r = −0,445 Chute e CS: r = −0,411 Arremesso e CS: r = 0,328 Agarrar e CS: r = −0,242	Associação negativa
Cliff et al.^32^, 2009	Processo; TGMD-2	Tempo total em CS; acelerômetro	Correlação de Pearson; CM e CS em meninos: r = −0,194 CM e CS em meninas: r = 0,138	Associação nula
Drenowatz e Greier^30^, 2019	Produto; DMT 6–18	Tempo de tela recreativo; questionário	Manova e correlação de Pearson Maior consumo de mídia com melhorias nos saltos laterais (p = 0,03) e diminuição na corrida de 6 minutos (p = 0,03)	Associação negativa
Famelia et al.^42^, 2017	Processo; TGMD-3	Tempo total em CS; acelerômetro	Regressão múltipla Habilidades de locomoção e CS durante o playground: r = −0,56 Habilidades com bola e CS durante o playground: r = −0,14	Associação incerta
Gu^43^, 2016	Produto; *PE Metrics*	Tempo total em CS; acelerômetro	Correlação de Pearson Habilidades de locomoção e CS: r = −0,13 Habilidades de controle de objetos e CS: r = −0,16 CM e CS: r = −0,19	Associação negativa
Gu et al.^44^, 2018	Produto; *PE Metrics*	Tempo total em CS; acelerômetro	Correlação de Pearson Habilidades de locomoção e CS: r = −0,30 Habilidades de controle de objetos e CS: r = −0,30 CM e CS: r = −0,34	Associação negativa
Gu, Chen e Zhang^45^, 2019	Produto; *PE Metrics*	Tempo total em CS; acelerômetro	Correlação de Pearson Habilidades de locomoção e CS em hispânicos: r = −0,25 Habilidades de controle de objetos e CS em hispânicos: r = −0,08 Habilidades de locomoção e CS em não hispânicos: r = −0,16 Habilidades de controle de objetos e CS em não hispânicos: r = −0,06	Associação negativa
Jaakkola et al.^46^, 2009	Produto; Arremesso, salto e equilíbrio	Tempo de tela recreativo; questionário	Correlação de Pearson Arremesso e CS: r = 0,09 Salto e CS: r = −0,28 Equilíbrio e CS: r = 0,22	Associação negativa
Jaakkola et al.^47^, 2019	Produto; Cinco saltos, arremesso e agarrar	Tempo total em CS; acelerômetro	Modelagem de equações estruturais 6º ano: Habilidades de locomoção e CS: r = −0,056 Habilidades de controle de objetos e CS: r = −0,142 7º ano: Habilidades de locomoção e CS: r = −0,364 Habilidades de controle de objetos e CS: r = −0,059	Associação incerta
Laukkanen et al.^48^, 2014	Produto; KTK e TCB	Tempo total em CS; acelerômetro	Correlação parcial CM e CS em pré-escolares meninos: r = −0,52	Associação incerta
Lloyd et al.^49^, 2014	Processo; TGMD	Tempo sentado; questionário	Correlação de Pearson Habilidades de locomoção e CS: r = −0,37 Habilidades de controle de objetos e CS: −0,10 CM e CS: r = −0,25	Associação negativa
Lopes et al.^33^, 2012	Produto; KTK	Tempo total em CS; acelerômetro	Regressão logística binária RP = 5,065 para meninas e RP = 9,149 para meninos	Associação negativa
Lopes et al.^50^, 2016	Produto; MABC-2	Tempo total em CS; acelerômetro	Correlação de Spearman Equilíbrio e CS: r = 0,15 Habilidades de controle de objetos e CS: r = 0,03	Associação nula
Matarma et al.^51^, 2018	Produto; BOT-2	Tempo total em CS; acelerômetro	Regressão linear Sem correlação significativa	Associação nula
Smith et al.^52^, 2015	Produto; arremesso, equilibrar em um pé, andar para trás	Tempo de tela recreativo; questionário	Regressão logística Alta CM e baixa probabilidade de alto tempo de tela aos 16 anos: RP = 0,79; IC 0,64–0,98 Alta CM e baixa probabilidade de tempo de TV aos 42 anos: RP = 0,85; IC 0,72–0,99	Associação negativa
Tsuda et al.^53^, 2019	Processo; TGMD-2	Tempo total em CS; acelerômetro	Correlação de Pearson Habilidades de locomoção e CS: r = −0,46 Habilidades de controle de objetos e CS: r = −0,42	Associação negativa
Webster et al.^17^, 2019	Processo e produto; TGMD-3 e MABC-2	Tempo total em CS; acelerômetro	Correlação de Pearson CM e tempo de tela: β = −1,6	Associação nula
Wrotniak et al.^37^, 2006	Produto; BOTMP-SF	Tempo total em CS; acelerômetro	Correlação de Pearson CM e CS: r = −0,308	Associação negativa

CM: competência motora; CS: comportamento sedentário; RP: razão de probabilidade; IC: intervalo de confiança; Manova: análise multivariada da variância; AST: *Athletic Skills Track*; BOT-2: *Bruininks-Oseretsky Test of Motor Proficiency Second Edition*; BOT-2 SF: *Bruininks-Oseretsky Test of Motor Proficiency Second Edition – Short Form*; BOTMP-SF: *Bruininks-Oseretsky Test of Motor Proficiency – Short Form*; DMT 6–18: *Deutsche Motorik Test*; KTK: *Körperkoordinationstest für Kinder*; MABC-2: *Movement Assessment Battery for Children Second Edition*; TCB: *Underarmor Throw and Catch a Ball*; TGMD: *Test of Gross Motor Development* [segunda e terceira edições assinaladas pelo número após a sigla].

Nota: Apenas os dados de crianças com desenvolvimento típico foram considerados para os resultados.

Quanto ao risco de viés, 18,2% (n = 4) dos estudos obtiveram uma pontuação de baixo risco (≥ 5), 77,3% (n = 17) obtiveram uma pontuação de médio risco e apenas um estudo obteve uma pontuação de alto risco. Todos os estudos atingiram os critérios: (a) “O estudo descreve os critérios de elegibilidade/seleção dos participantes?” e (d) “O estudo menciona fontes e detalhes da avaliação do CS?”. Finalmente, os itens de qualidade que estiveram mais ausentes foram: (b) “Os participantes foram selecionados aleatoriamente?” e (e) “O estudo relatou dimensionamento amostral e foi adequadamente dimensionado para detectar relações hipotéticas?” (Figura 2).

**Figura 2:** Análise por risco de viés dos artigos incluídos.

Do total de estudos investigados (n = 22), treze apontaram associações negativas entre CS e CM, seis não apontaram associação e três apontaram associações incertas.

No estrato de estudos avaliados com baixo risco de viés (n = 4), foi encontrada associação negativa em três estudos, destacando como principais resultados: CS e alta CM em escolares^12^; CS e CM em pré-escolares^44^; CM na infância e tempo sentado após 20 anos^49^. Resultados de associação negativa também foram encontrados em estudos com risco de viés moderado^30,33,37,40,41,43,45,46,52,53^.

Considerando os estudos experimentais (n = 2) de médio risco de viés, um estudo^41^ encontrou associação negativa entre CS e CM nas habilidades de locomoção e controle de objetos, porém o outro^48^ encontrou associação incerta. Considerando os estudos longitudinais (n = 9), dois estudos de baixo risco de viés encontraram associação negativa entre CS e CM^44,49^, assim como outros cinco estudos de médio risco de viés^30,40,43,45,52^ encontraram associação negativa entre CS e CM. Em contraste, um estudo de alto risco de viés^51^ não encontrou associação e um estudo de moderado risco de viés^47^ encontrou associação incerta. Considerando os estudos transversais (n = 11) um estudo de baixo risco de viés encontrou associação negativa entre CS e CM^12^ e quatro estudos de médio risco de viés^33,37,46,53^ encontraram associação negativa entre CS e CM. Em contraste, um estudo de baixo risco de viés^39^ e quatro estudos de médio risco de viés^17,32,38,50^ não encontraram associação, e um estudo de médio risco de viés^42^ encontrou associação incerta.

Analisando a faixa etária de pré-escolares (n = 11), dois estudos de baixo risco de viés^44,49^ e três estudos de médio risco de viés^40,43,53^ encontraram associação negativa entre CS e CM. Em contraste, um estudo de baixo risco de viés^39^, três estudos de médio risco de viés^17,32,50^ e um estudo de alto risco de viés^51^ não encontraram associação, enquanto um estudo encontrou associação incerta^42^. Sendo assim, de acordo com os critérios estabelecidos, os resultados demonstram evidência incerta de associação entre CS e CM nessa faixa etária. Considerando a faixa etária de ensino fundamental (n = 11), um estudo de baixo risco de viés^12^ e sete estudos^30,33,37,41,45,46,52^ de médio risco de viés encontraram associação negativa entre CS e CM. Em contraste, um estudo de médio risco de viés^38^ não encontrou associação e dois estudos encontraram associação incerta. Portanto, os resultados indicam evidências de associação negativa entre CS e CM nesta faixa etária (Tabela 3).

**Tabela 3:** Distribuição dos estudos que investigaram comportamento sedentário e competência motora pelo risco de viés dentro dos estudos e pelo nível de evidência científica.

Escolaridade	Estudos que demonstraram associação	Estudos por risco de viés	Associação ou não associação de acordo com o risco de viés	Nível de evidência
Pré-escola (n = 11)	Associação negativa: 5 (45,4%) Não associação: 5 (45,4%) Associação incerta: 1 (9,1%)	Baixo: 3 (27,2%) Médio: 7 (63,6%) Alto: 1 (9,0%)	BRV: N:^44,49^; NA:^39^ MRV: N:^40,43,53^; NA:^32,50,17^; ?: ^42^ ARV: NA:^51^	Evidência incerta
Ensino fundamental (n = 11)	Associação negativa: 8 (88,8%) Não associação: 1 (9,0%) Associação incerta: 2 (18,1)	Baixo: 1 (9,0%) Médio: 10 (90,9%)	BRV: N:^12^ MRV: N:^41,30,45,46,33,52,37^; NA:^38^; ?:^47,49^	Evidência de associação negativa

BRV: baixo risco de viés; MRV: médio risco de viés; ARV: alto risco de viés; N: associação negativa; NA: associação nula; ?: associação incerta.

Em suma, considerando o resultado das associações e o risco de viés, houve evidência científica para a associação negativa entre CS e CM de crianças e adolescentes do ensino fundamental e evidência científica incerta para associação em crianças pré-escolares.

## DISCUSSÃO

A presente revisão sintetizou os resultados dos estudos que avaliaram associações entre CS e CM na infância e adolescência. De acordo com os resultados, pode-se afirmar que há evidência para associação negativa entre CS e CM nos anos do ensino fundamental. Mesmo que a maioria dos estudos examinados tenha usado um delineamento transversal, dificultando inferências sobre causalidade, sete estudos longitudinais apontaram associações negativas entre CS e CM, sugerindo que o tempo em CS pode prejudicar o desenvolvimento da CM^30,40,43^.

A metanálise de Engel et al.^54^ sugere que a CM e os níveis de AF podem aumentar por meio de intervenções na infância, corroborando os achados de uma revisão prévia^55^, na qual as habilidades de controle de objetos foram fortemente associadas à AF em meninos, e as habilidades de locomoção foram associadas à AF em meninas. Um recente estudo longitudinal demonstrou a diminuição de AF e aumento do CS entre 6 e 11 anos^56^. Uma hipótese plausível, baseada nos resultados encontrados no presente estudo e no modelo conceitual proposto por Stodden et al.^24^, é que a CM adequada poderia influenciar o aumento da AF e a diminuição do CS. É possível que crianças com excesso de CS tenham poucas oportunidades de desenvolvimento da CM^30^, aumentando ainda mais a probabilidade de adoção de CS (atividades de tela) nos momentos de lazer^18,29^ e gerando um ciclo comportamental vicioso com hábitos de vida não saudáveis associados ao surgimento de doenças crônico-degenerativas^24^. Assim, é importante que políticas públicas de saúde considerem a relação recíproca e dinâmica entre CS e CM e promovam, simultaneamente, a melhora da CM e a diminuição do CS, particularmente do tempo de lazer em atividades de tela durante a infância e adolescência.

Quanto aos fatores ambientais, o modelo teórico de Hulteen et al.^57^ sugere que as habilidades motoras variam em importância e popularidade de acordo com o contexto cultural e geográfico, com papel importante para a manutenção da AF ao longo da vida. Considerando o papel da atividade física como forma de intervenção para diminuir o CS e contribuir para o desenvolvimento da CM, é possível sugerir que quanto maior a CM em diferentes habilidades (por exemplo, chutar, arremessar, quicar e saltar), maior a gama de possibilidades de prática de AF que poderiam substituir o CS. O desenvolvimento da CM em habilidades que predominam no contexto cultural e geográfico em questão pode ser uma estratégia eficaz para substituir o CS por uma AF que a criança ou adolescente tenha competência para executar. Reforçando essa perspectiva, dados de um estudo finlandês^41^ apontam associação negativa entre a habilidade do chute e o CS, sendo o futebol um dos esportes juvenis predominantes no país^58^.

Os dados desta revisão reforçam a importância de políticas públicas integradas de educação e saúde focadas, simultaneamente, na melhoria da CM e diminuição do CS, particularmente do tempo de tela no lazer. Intervenções em crianças que passam tempo excessivo em CS devem incluir o desenvolvimento da CM de forma contínua, ou seja, deve-se levar em consideração a qualidade da prática de AF, não apenas a quantidade de tempo dispendida nessa prática^59,60^. A melhora da CM pode ser uma estratégia promissora para reduzir o CS e aumentar a AF moderada a vigorosa, principalmente para as crianças com baixa CM^12^. É importante que futuras pesquisas sejam fortalecidas por avaliações longitudinais para proporcionar melhor compreensão da causalidade entre CS e CM.

Além disso, existe vasta margem a ser explorada, levando em consideração fatores ambientais, gênero e métodos de avaliação, bem como a lacuna existente na transição entre faixas etárias (crianças, adolescentes, adultos jovens e idosos). É plausível que a direção causal entre as variáveis seja influenciada pela faixa etária. Recomendam-se para futuros estudos a utilização de dois ou mais testes motores^22,61^ e o uso do acelerômetro conjuntamente ao questionário para o melhor entendimento do CS e fatores ambientais. Sugerem-se também a seleção randomizada dos participantes e descrição do poder estatístico do estudo. Por fim, também é importante destacar que os resultados encontrados se limitam aos adolescentes até 14 anos, proporcionando vasto campo de pesquisa entre adolescentes e jovens adultos nesta temática.

O presente estudo contém algumas limitações. Embora a busca tenha sido abrangente, apenas estudos na língua inglesa foram incluídos. O viés relacionado ao relato seletivo das associações nos estudos pode ser uma possibilidade, e não foram considerados os ajustes nas associações entre CS e CM em função da prática de AF. Além disso, os resultados desta revisão são influenciados pelas limitações na base de evidências. Os efeitos negativos do CS podem ser compensados parcialmente pela prática de AF moderada a vigorosa (AFMV)^31,62^, sendo comportamentos independentes, ou seja, altos níveis de CS não necessariamente implicam em baixos níveis de AFMV e vice-versa; pessoas que atendem às recomendações de AFMV (≥ 1h diária) ainda podem ter CS em muitas horas do dia^63^. Na realidade, até o momento, as recomendações de CS são restritas ao tempo de tela^1,15,64^. Do ponto de vista matemático, se for aplicada a recomendação de no mínimo três horas diárias de AF em qualquer intensidade (incluindo 1h de AFMV)^1,15^, e considerando um período de 12h de vigília, o tempo limite de CS seria de 9h diárias. Portanto, é necessário um maior entendimento do contexto em que o CS se manifesta e sua associação com o desenvolvimento global da criança ou adolescente.

Outra limitação é a heterogeneidade nas formas utilizadas para avaliar o CS^65^ (questionários com informações sobre o tempo total de tela ou somente tempo de TV/computador ou tempo sentado e medidas diretas de acelerometria), impossibilitando uma diferenciação do CS em atividades de tela/lazer e do CS em outros domínios, como tempo sentado no deslocamento ou nos estudos. O uso de diferentes testes motores para aferir a CM também é uma limitação importante. No total, pelo menos 10 avaliações diferentes de CM foram utilizadas. Na literatura a CM pode ser avaliada por meio de medidas orientadas ao processo ou ao produto do movimento, e isso pode influenciar a magnitude das associações^22^.

Finalmente, os resultados desta revisão sistemática demonstram que há evidências de associação negativa entre CS e CM em crianças e adolescentes no período do ensino fundamental, embora a evidência seja incerta nos anos pré-escolares. Os fatores ambientais, assim como o método de avaliação e faixa etária, podem ser determinantes para a melhor compreensão do fenômeno investigado. Para o melhor entendimento das associações entre CS e CM, sugerimos o estabelecimento de critérios padronizados para a condução dos estudos, destacando o contexto em que o CS se manifesta (por exemplo, em situações de lazer ou atividades escolares). Melhorar a CM predominante no contexto sociocultural em que a pessoa está inserida pode contribuir para a diminuição do CS e promover o engajamento em um estilo de vida ativo em longo prazo para as crianças e adolescentes.
